# A Network Pharmacology Approach to Explore the Mechanisms of *Artemisiae scopariae* Herba for the Treatment of Chronic Hepatitis B

**DOI:** 10.1155/2021/6614039

**Published:** 2021-02-02

**Authors:** Asi He, Wei Wang, Yang Xia, Xiaoping Niu

**Affiliations:** Department of Gastroenterology, Yijishan Hospital of Wannan Medical College, Wuhu 241000, China

## Abstract

**Background:**

As a traditional Chinese medicine, *Artemisiae scopariae* Herba (ASH) is used to treat various liver diseases. The purpose of this study was to explore the mechanisms of ASH for treating chronic hepatitis B (CHB) using a network pharmacological method.

**Methods:**

Bioactive ingredients and related targets of ASH were obtained from Traditional Chinese Medicine Systems Pharmacology (TCMSP) database. Gene names of targets were extracted from UniProt database. Differentially expressed genes (DEGs) of CHB were obtained from microarray dataset GSE83148. The intersect genes between DEGs and target genes were annotated using clusterProfiler package. The STRING database was used to obtain a network of protein-protein interactions. Cytoscape 3.7.2 was used to construct the “ingredient-gene-pathway” (IGP) network. Molecular docking studies were performed using Autodock vina.

**Results:**

A total of 13 active components were extracted from TCMSP database. Fifteen intersect genes were obtained between 183 target genes and 403 DEGs of GSE83148. Kyoto Encyclopedia of Genes and Genomes (KEGG) enrichment analysis results showed that ASH against CHB mainly involved in toll-like receptor signaling pathway, cellular senescence, hepatitis B, and chemokine signaling pathway. We screened one hub compound, five core targets, and four key pathways from constructed networks. The docking results indicated the strong binding activity between quercetin and AKT1.

**Conclusions:**

This study provides potential molecular mechanisms of ASH against CHB based on exploration of network pharmacology.

## 1. Introduction

Chronic hepatitis B (CHB) is a chronic liver inflammatory disease caused by persistent infection of hepatitis B virus (HBV). Despite the availability of effective vaccines, HBV still affects more than 250 million people worldwide [1]. The majority of individuals with HBV infection are clinically asymptomatic; however, persistent infection could result in variable liver diseases, including hepatitis, liver cirrhosis, and hepatocellular carcinoma [2, 3]. Currently, oral nucleoside reverse transcriptase inhibitors and interferon (IFN) alpha are available effective antiviral drugs for treatment of chronic HBV infection [4]. However, with challenges in medication cost, drug resistance mutation, and toxicity, it is necessary to explore new treatment drugs and targets in anti-HBV.

Yin-Chen (Latin name: *Artemisiae scopariae* Herba, ASH), the main constituents of which include coumarins, flavonoids, organic acids, and volatile oils, is a traditional Chinese medicine (TCM). ASH has multiple biological activities, such as liver protection, antivirus, antitumor, neuroprotection, prevention of Alzheimer's disease, and metabolic regulation [5]. Previous studies have confirmed that ASH can inhibit the secretion of HBsAg and HBeAg and HBV DNA replication [6–8]. Furthermore, clinical studies have shown that Yinchenhao decoction can significantly improve CHB patients' clinical symptoms and restore their liver function [9, 10]. However, the pharmacological mechanisms of ASH treatment of CHB are not completely clear.

**Figure 1:**
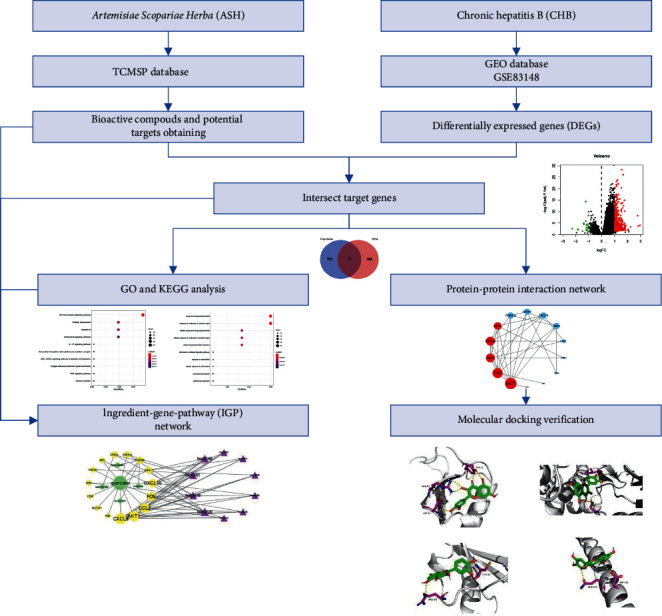
Workflow chart of *Artemisiae scopariae* Herba in the treatment of chronic hepatitis B based on network pharmacology.

TCMs are multicomponent, multichannel, and multitarget, showing advantages in the treatment and prevention of diseases [11]. With the comprehensive use of the methods of network biology, bioinformatics, and chemical informatics, network pharmacology can systematically study and explore the pharmacological mechanism and effective substances of TCMs. It has become an effective means to discover active substances and reveal the pharmacological mechanism of TCMs [12]. In this study, we investigated the mechanisms on how ASH exerts its therapeutic effects on CHB using strategy of network pharmacology, which may provide a basis for further experimental study. The workflow plot is shown in Figure 1.

## 2. Materials and Methods

### 2.1. Bioactive Compounds and Target Genes of ASH

The bioactive compounds of ASH were obtained from the TCMSP database (http://www.tcmspw.com/tcm.php, Version 2.3) [13]. The effective compounds were screened by pharmacokinetic absorption, distribution, metabolism, and excretion (ADME) of criterion: oral bioavailability (OB) ≥30% and drug-likeness (DL) ≥0.18 [14, 15]. Targets of effective compounds were gained from the TCMSP database, and the target proteins corresponding to effective compounds were standardized in UniProt (http://www.uniprot.org/). The structures of effective compounds were obtained from PubChem (https://pubchem.ncbi.nlm.nih.gov/).

### 2.2. Gene Expression Profiles

Microarray datasets GSE83148 were downloaded from Gene Expression Omnibus (GPL570 Affymetrix Human Genome U133 Plus 2.0 Array). These datasets consisted of 122 HBV infected liver tissues and 6 healthy controls. Differentially expressed genes (DEGs) were obtained using Limma package with adjusted *p* value <0.05 and |log_2_FoldChange| > 1. The intersect genes were identified between target genes and DEGs using Venny online tool (http://bioinformatics.psb.ugent.be/webtools/Venn/).

### 2.3. Functional Enrichment Analysis and Network Construction

Gene ontology (GO) and Kyoto Encyclopedia of Genes and Genomes (KEGG) analyses were implemented using the “clusterProfiler” package. The intersect genes were imported into STRING database (http://string-db.org/) for the protein interaction analysis (the combined score >0.4) [16], and the Cytoscape software 3.7.2 (http://www.cytoscape.org) was performed to construct the PPI network. Five effective ingredients, fifteen intersect genes, and the top 10 KEGG pathways were also introduced into Cytoscape to establish a visualized ingredient-gene-pathway (IGP) network. The tool of network analyzer was used to calculate topological parameters. Three topological parameters including degree centrality (DC), betweenness centrality (BC), and closeness centrality (CC) were used to estimate the central properties of the nodes in PPI and IGP network [17]. DC ≥ median DC, BC ≥ median BC, and CC ≥ median CC were employed to screen the core targets, hub genes, and key pathways [17]. The correlation between hub genes was determined by Pearson's analysis.

### 2.4. Molecular Docking Study

The structures of core targets, quercetin, and positive control drug (entecavir) were obtained from PDB (http://www.pdb.org/) and PubChem, respectively. Docking studies were performed between core targets and quercetin or entecavir using Autodock vina (Version, 1.1.2) [18]. The docking results were visualized using Pymol v.2.3 software, and docking effects were evaluated by the affinity value (AV). The AV of entecavir was used as the baseline. The AV >−5, ≤−5, ≤−7, and ≤−9 kcal/mol mean no, certain, good, and strong binding activity, respectively [19, 20].

## 3. Results

### 3.1. Potential Bioactive Ingredients and Drug Genes

A total of 13 bioactive compounds of ASH were obtained from the TCMSP database, including 4′-methylcapillarisin, areapillin, beta-sitosterol, isorhamnetin, skrofulein, isoarcapillin, eupalitin, eupatolitin, capillarisin, demethoxycapillarisin, quercetin, artepillin A, and genkwanin. 2D structures of these compounds were extracted from PubChem Database (see Table 1). We got 360 target proteins from the TCMSP database and used UniProt database to obtain corresponding target genes. After removing the duplicates, 183 target genes were identified as drug genes.

### 3.2. Intersect Genes

**Figure 2:**
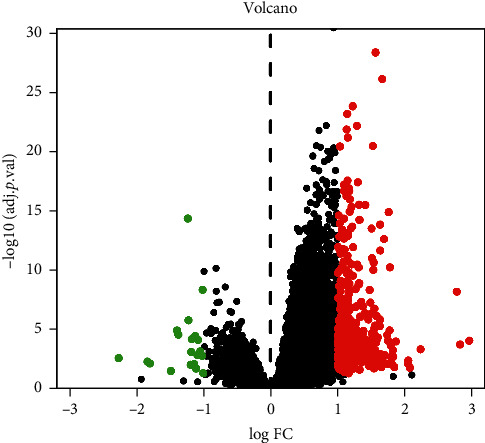
Differentially expressed genes (DEGs) in volcano plot. Green and red dots indicate downregulated and upregulated genes, respectively, on the basis of |log2FoldChange| > 1 and adjusted *p* value <0.05.

**Figure 3:**
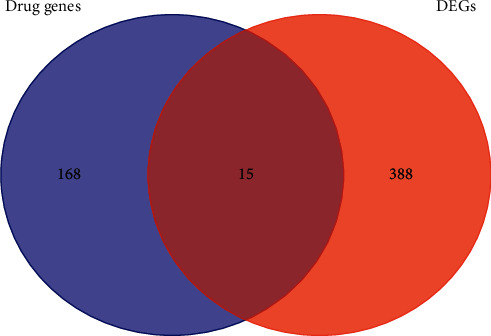
Venn diagram of drug genes (blue) and differentially expressed genes (red).

A total of 403 DEGs were screened from GSE83148 and visualized by a volcano plot (see Figure 2), 383 of which were upregulated and 20 were downregulated. Then, we used Venny online tool to get 15 intersect genes between drug genes and DEGs (see Figure 3).

### 3.3. Enrichment Analysis

**Figure 4:**
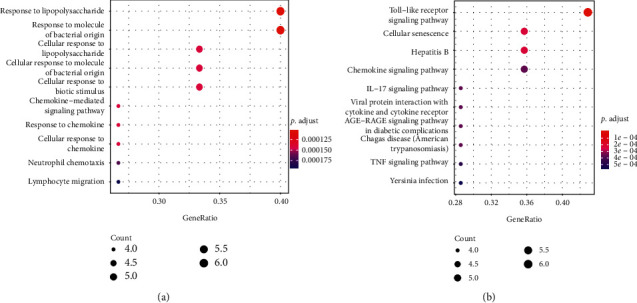
Functional annotation of 15 intersect genes by (a) biological process and (b) KEGG pathway.

GO analysis showed that 15 intersect genes mainly participated in response to lipopolysaccharide, cellular response to lipopolysaccharide, cellular response to biotic stimulus, chemokine-mediated signaling pathway, response to chemokine, cellular response to chemokine, neutrophil, chemotaxis, and lymphocyte migration. KEGG analysis showed that intersect genes involved in toll-like receptor (TLR) signaling, cellular senescence, hepatitis B, chemokine signaling pathway, IL−17 signaling pathway, viral protein interaction with cytokine and cytokine receptor, and TNF signaling pathway (see Figure 4). These results suggested that ASH may play a pharmacological role in treatment of CHB by regulating inflammation-related pathways, immunomodulation-related pathways, and viral infection-related pathways.

### 3.4. Network Analysis

**Figure 5:**
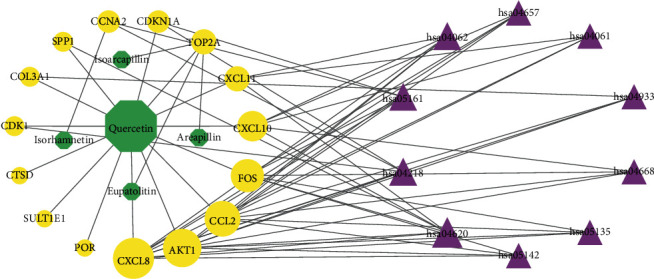
Ingredient-gene-pathway network. The purple triangles, yellow ellipses, and green octagons represent pathways, intersect target genes, and effective ingredients of ASH, respectively. The larger the shape of the graph, the greater the degree value of the node, and the greater the role in the network.

**Figure 6:**
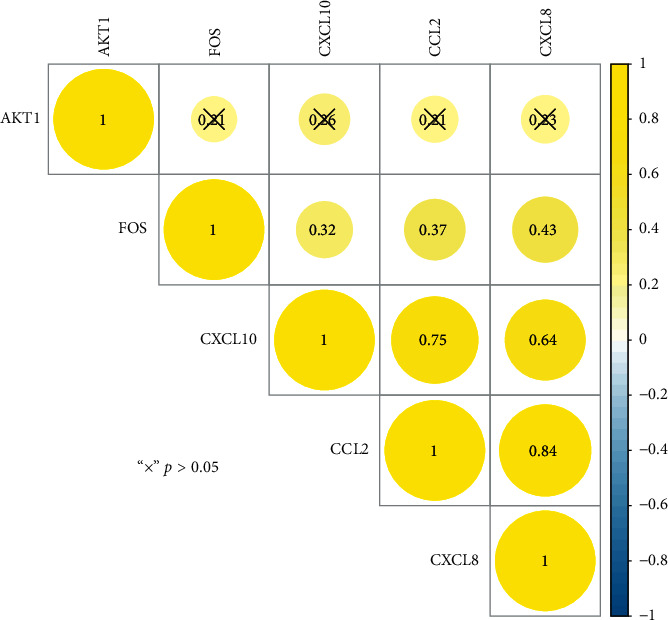
Pearson's correlation analysis of the hub genes in GSE83148. Yellow indicates significant positive correlation.

**Figure 7:**
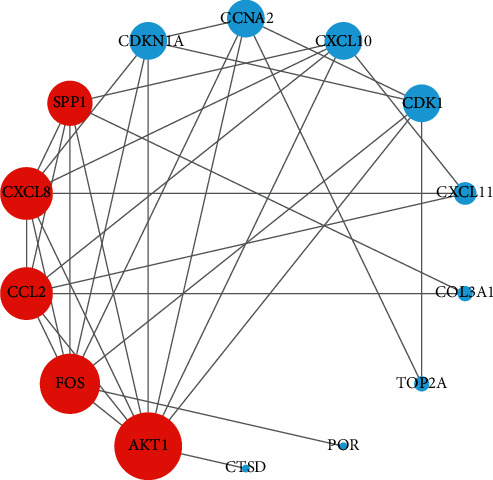
Target protein interaction network and hub targets analysis. The nodes and edges indicate proteins and protein-protein associations, respectively. Red nodes represent core proteins. The larger the round shape, the greater the degree value of the node.

The IGP network comprised 30 nodes (5 effective components, 15 intersect genes, and 10 pathways) and 63 edges (see Figure 5). In this network, we found that 14 out of 15 intersect genes are connected to quercetin. This result suggested that quercetin may be the hub active ingredient of ASH against CHB. Topological analysis indicated that AKT1, FOS, CCL2, CXCL8, and CXCL10 are hub genes; TLR signaling pathway, cellular senescence, hepatitis B, and chemokine signaling pathway are key pathways in the IGP network. In addition, we analyzed the correlation among hub genes in GSE83148 datasets (see Figure 6). These hub genes were significantly upregulated in HBV infected liver tissues compared with healthy control and had a significant positive correlation with each other except for AKT1. Among them, CXCL8 and CCL2 had the highest correlation with each other. In the PPI network, we screened five core targets: RAC-alpha serine/threonine-protein kinase (AKT1), proto-oncogene c-Fos (FOS), C-C motif chemokine 2 (CCL2), osteopontin (SPP1), and interleukin-8 (CXCL8) (see Figure 7).

### 3.5. Molecular Docking Validations

**Figure 8:**
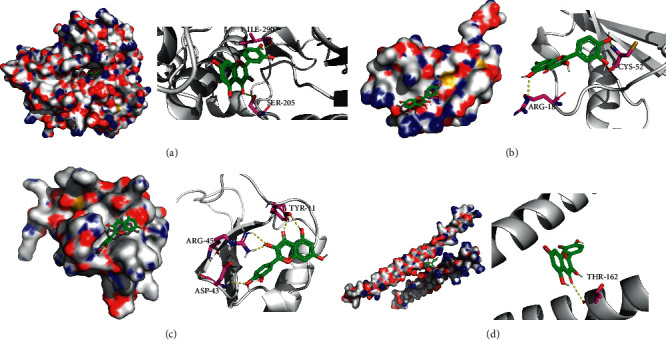
Molecular docking diagram. Molecular models of the binding of AKT1, CCL2, CXCL8, and FOS with quercetin, the results shown as 3D diagrams. (a) Quercetin-AKT1 (AV = −9.3 kcal/mol); (b) quercetin-CCL2 (AV = −5.8 kcal/mol); (c) quercetin-CXCL8 (AV = −6.6 kcal/mol); (d) quercetin-FOS (AV = −6.1 kcal/mol).

We did not adopt the SPP1 in a molecular docking study because 3D structure of SPP1 did not have a human source in the PDB database. We docked quercetin and entecavir with four of above core targets and found quercetin had certain binding activity with CCL2, FOS, and CXCL8 (AV ≤ −5 kcal/mol) and strong binding activity with AKT1 (AV ≤ −9 kcal/mol) (see Table 2). Interestingly, the AV of key targets binding with quercetin was lower than that of entecavir. These results suggested that AKT1, CCL2, CXCL8, and FOS may be the key targets for the pharmacological action of quercetin (see Figure 8).

## 4. Discussion

In the IGP network, we identified five active compounds, including quercetin, eupatolitin, isorhamnetin, areapillin, and Isoarcapillin. Among them, quercetin had the largest node value, indicating that quercetin played a pivotal role in the network. Quercetin is classified as flavonol, which possesses various biological and pharmacological activities including antioxidative, antiviral, anti-inflammatory, and antifibrotic effects [21, 22]. Parvez et al. reported that quercetin may exert anti-HBV activity through binding with HBV polymerase and capsid protein as well as host sodium taurocholate cotransporting polypeptide proteins [23]. Li et al. reported that quercetin diminished carbon tetrachloride-induced liver inflammation and fibrosis in mice through inhibiting macrophages infiltration and modulating M1 macrophages polarization via targeting Notch1 pathway [22]. In this IGP network, we obtained 5 hub genes, including AKT1, FOS, CCL2, CXCL8, and CXCL10, which were connected to quercetin; in addition, TOP2A was linked to multiple effective compounds. These findings suggested that these genes may be target genes of ASH against CHB. In the IGP network, the pattern of effective compound interacting with target genes was consistent with the modern drug multicomponent and multitarget theory: one effective compound interacted with multitarget genes and a target gene interacted with multieffective compounds [24].

In PPI network, we ascertained 5 core targets: AKT1, FOS, CCL2, SPP1, and CXCL8. Then, we used quercetin as a ligand to dock with the core protein. The results of molecular docking showed that AKT1, CXCL8, CCL2, and FOS had binding activity to quercetin, which suggested that the above proteins may be the key target proteins for the pharmacological action of ASH. Prior study has reported CXCL8 and its receptor CXCR1 are upregulated intrahepatically in chronic liver diseases [25]. In addition, CXCL8 could be induced by HBV directly and induces HBV replication in turn contributing to the persistence of infection [26]. IL-8 may help reduce the sensitivity of HBV to IFN-alpha and damage the activity of IFN-alpha in inhibiting HBV replication [26]. It has been reported that the expression of CCL2/MCP-1 in CHB patients is significantly increased, which is positively correlated with the inflammatory degree of liver tissue [27]. CCL2 could recruit CCR2^+^Ly-6C^hi^ monocytes to develop into Ly-6C^+^ macrophages in the damaged liver; then, these Ly-6C^+^ macrophages provoked hepatic stellate cells (HSCs) and promoted hepatic fibrosis in mice [28, 29]. Furthermore, Baeck et al. reported that CCL2-inhibitor diminished liver fibrosis by suppressing Ly-6C^+^ macrophages infiltration in mice [30]. Some researchers reported that PI3K and Akt played an important role in the activation and proliferation of the collagen synthesis of HSCs in mice [31, 32]. Another study found that both Akt1 and Akt2 promoted proliferation of HSCs and participated in the fibrogenesis in mice liver [33]. Sun et al. reported that decreased AKT protein levels in HepG2.2.15 cells were associated with decreased HBV replication in a time-dependent manner [34]. c-Fos was one member of the dimeric activator protein 1 transcription factor family that regulated key cellular processes, including cell death, proliferation, and differentiation [35, 36]. Kalra and Kumar demonstrated that c-Fos-induced apoptosis through activation of c-Myc in hepatoma cells and hepatitis B virus X protein not only inhibited the expression of c-Fos but also transactivated c-Fos promoter [37]. These findings suggested that c-Fos may participate in the processes of apoptosis of liver cells and HBV self-replication. We speculate that ASH may play an antifibrosis and antiviral effect by regulating the abovementioned targets in patients with CHB.

KEGG pathway analysis showed that the intersect genes were enriched in the top 10 pathways as follows: TLR signaling pathway, cellular senescence, hepatitis B, chemokine signaling pathway, IL-17 signaling pathway, viral protein interaction with cytokine and cytokine receptor, and TNF signaling pathway. Then, we identified four key signaling pathways in the IGP network: TLR signaling pathway, cellular senescence, hepatitis B, and chemokine signaling pathway. Some studies have shown that these key pathways are involved in the pathogenesis of CHB. Isogawa et al. reported that TLR signaling inhibited HBV replication by trigging the production of cytokines (such as IFN-alpha and IFN-gamma) in mice liver [38]. Previous studies have reported that TLR agonists may be used as immunomodulators in the treatment of CHB by enhancing HBV-specific T cell responses [39–41]. Some other studies also confirmed that the senescence of activated hepatic stellate cells contributed to the reversal of hepatic fibrosis [42–44]. Tachtatzis et al. reported that HBV infection mediate G1 arrested hepatocytes and induced hepatocyte senescence [45]. In this study, AKT1 and CXCL8 are enriched in hepatitis B (hsa05161), which may be involved in the regulation of HBV replication [26, 46]. Chemokine signaling pathway plays an important role in human liver inflammation and fibrosis [47, 48]. Inhibition of the CCR2-CCL2 axis promotes improvement of fibrosis in a variety of fibrotic animal models, suggesting that CCL2-CCR2 axis may be a target for drug intervention [29, 49, 50]. In addition, CCL5 and its related CCR5 ligand CCL3 and CCL4 are upregulated in the liver of patients with fibrosis. Inhibition of CCL5 may accelerate the regression of liver fibrosis through inhibiting function of HSCs and balancing monocyte/macrophage subsets [51–53]. Therefore, we found that these signal pathways were closely related to CHB, and ASH played a role in the treatment of CHB by regulating these signal pathways.

There are some defects in present work. This study lacks experimental verification, which will be further validated in future study. The composition of ASH is complex and some components or related prediction targets may be missed due to incomplete database information and slow update.

## 5. Conclusions

This study reflects the characteristics of “multicomponent, multitarget, and multipathway” of ASH in the treatment of CHB. We identified quercetin acted on the targets (AKT1, FOS, CCL2, and CXCL8) through key signal pathways (toll-like receptor signal pathway, hepatitis B, cell senescence, and chemokine signaling pathway) to exert treatment effects on CHB by use of a network pharmacological method. This study provides clues for understanding the molecular mechanisms of potential targets of ASH in the treatment of CHB and provides a reference for the further experimental study.

## Figures and Tables

**Table 1:** Active ingredients and ADME parameters of *Artemisiae scopariae* Herba (ASH).

No.	Molecule ID	Molecule name	Chemical formula	Structure	OB (%)	DL
1	MOL000354	Isorhamnetin	C_16_H_12_O_7_	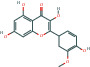	49.6	0.31
2	MOL000358	Beta-sitosterol	C_29_H_50_O	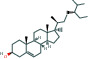	36.91	0.75
3	MOL004609	Areapillin	C_18_H_16_O_8_	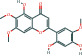	48.96	0.41
4	MOL005573	Genkwanin	C_16_H_12_O_5_	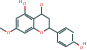	37.13	0.24
5	MOL007274	Skrofulein	C_17_H_14_O_6_	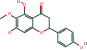	30.35	0.3
6	MOL008039	Isoarcapillin	C_18_H_16_O_8_	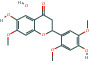	57.4	0.41
7	MOL008040	Eupalitin	C_17_H_14_O_7_	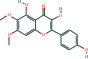	46.11	0.33
8	MOL008041	Eupatolitin	C_17_H_14_O_8_	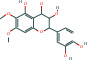	42.55	0.37
9	MOL008043	Capillarisin	C_16_H_12_O_7_	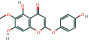	57.56	0.31
10	MOL008045	4′-Methylcapillarisin	C_17_H_14_O_6_	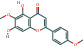	72.18	0.35
11	MOL008046	Demethoxycapillarisin	C_15_H_10_O_6_	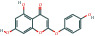	52.33	0.25
12	MOL008047	Artepillin A	C_19_H_24_O_4_	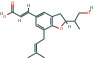	68.32	0.24
13	MOL000098	Quercetin	C_15_H_10_O_7_	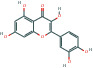	46.43	0.28

ADME: absorption, distribution, metabolism, and excretion.

**Table 2:** Docking scores of the quercetin and entecavir with potential targets.

Targets	PDB ID	Compound	Affinity (kcal/mol)
AKT1	6HHJ	Quercetin	−9.3
AKT1	6HHJ	Entecavir	−8
CCL2	3IFD	Quercetin	−5.8
CCL2	3IFD	Entecavir	−5.5
CXCL8	6N2U	Quercetin	−6.6
CXCL8	6N2U	Entecavir	−5.7
FOS	1FOS	Quercetin	−6.1
FOS	1FOS	Entecavir	−5.7

## Data Availability

All data are available from the corresponding author upon reasonable request.
